# 4239 Racial/ethnic difference in the relationship between periodontitis and cardiovascular disease among adult populations

**DOI:** 10.1017/cts.2020.422

**Published:** 2020-07-29

**Authors:** Magda Shaheen, Neeraj Harish Khona, Katrina Schrode

**Affiliations:** 1 David Geffen School of Medicine at UCLA; 2 Charles R Drew University

## Abstract

OBJECTIVES/GOALS: Several lines of studies have supported the existence of periodontitis (inflammation of the gums) as a risk factor for cardiovascular disease (CVD). The goal of this study is to evaluate the relationship between periodontitis and CVD among Hispanic, African American, and Caucasian populations. METHODS/STUDY POPULATION: We analyzed data from the National Health and Nutrition Examination Survey, 1999-2004 (NHANES). The population was all adults with a periodontal exam. Periodontal Disease was defined as mild, moderate, and severe (2 loss of attachments of at least 3mm, 2 sites with probing depth of at least 4mm, or one site with probing depth of at least 5mm). Cardiovascular disease was defined by a questionnaire regarding prevalence of any of 5 diagnosis (congestive heart failure [CHF], coronary artery disease [CAD], angina, heart attack or stroke). Data were analyzed using multinomial regression in SAS version 9.3 taking into consideration the design and weight. RESULTS/ANTICIPATED RESULTS: The study included 3375 adults; 13% were Hispanic and 10% were Blacks, 58% had > high school education, 81% were insured, 11% were heavy alcohol drinkers, 27% were smokers, 13% were physically inactive, 14% had periodontitis, 62% visited dentist last year, 2% had CHD, and 1.5% had CHF or stroke. In the multiple multinomial regression, overall, people with periodontitis were more likely to have both CHD (AOR = 2.0, 95% CI = 1.1-3.8, p<0.05) and CHF or stroke (AOR = 1.8, 95% CI = 1.01, 3.0, p<0.05) than to have no heart condition. There was a racial/ethnic difference in the relationship between periodontitis and cardiovascular disease but it was not statistically significant (p>0.05). DISCUSSION/SIGNIFICANCE OF IMPACT: Overall, people with periodontitis were more likely to have CHD, CHF or stroke than to have no heart condition, but with no significant effect of racial/ethnic group. This study provides a foundation to future studies on the connection of periodontitis and CVD in relation to ethnic/racial groups.

